# Exploring Mild Cognitive Impairment and Cognitive Rehabilitation in a cohort from the Alzheimer Center of the School of Medicine of the University of Buenos Aires

**DOI:** 10.1002/alz.093342

**Published:** 2025-01-09

**Authors:** Natividad Olivar, Maria Dolores Barreto, Vanesa Roxana Mendez, Patricia Alejandra Pasqua, Karina Irene Estigarribia, Luis Ignacio Brusco

**Affiliations:** ^1^ ALZAR ‐ Argentine Alzheimer’s Association, Buenos Aires, CABA Argentina; ^2^ University of Buenos Aires, Buenos Aires Argentina; ^3^ Clincal Hospital, School of Medicine, University of Buenos Aires, Buenos Aires Argentina; ^4^ National Scientific and Technical Research Council (CONICET), Buenos Aires Argentina

## Abstract

**Background:**

Mild cognitive impairment (MCI) is a condition that affects a growing number of individuals worldwide, representing a transition between normal aging and dementia. As the world’s population ages, understanding and addressing MCI becomes increasingly crucial for both patients and healthcare professionals.

**Method:**

15 participants (13 women and 2 men) with MCI with an MMSE score of 24/30 were included in a cognitive rehabilitation workshop. The workshop was held during the year 2023, with a frequency of twice a week. A neuropsychological battery was applied to each participant at the beginning and at the end of the workshop with the following tests: semantic and phonological verbal fluency, Trail A and B tasks, Boston test, Digit‐symbol test and the Rey’s verbal test. The Beck Depression Inventory and the Katz and Lawton and Brody scales were also administered to evaluate activities of daily and instrumental living.

**Result:**

73% of patients showed an improvement in MMSE scores. They also showed similar but significantly better performance in the neuropsychological tests administered. 66% showed a reduction in abnormally high Beck Depression Inventory scores, while activities of daily living and instrumental living improved.

**Conclusion:**

Early and group intervention can help mitigate the effects of MCI and promote healthy cognitive aging by improving sociability, mood, and quality of life.

